# Electroacupuncture Inhibits NLRP3 Activation by Regulating CMPK2 After Spinal Cord Injury

**DOI:** 10.3389/fimmu.2022.788556

**Published:** 2022-03-24

**Authors:** Yi Chen, Lei Wu, Mengting Shi, Danyi Zeng, Rong Hu, Xingying Wu, Shijun Han, Kelin He, Haipeng Xu, XiaoMei Shao, Ruijie Ma

**Affiliations:** ^1^ Department of Neurobiology and Acupuncture Research, The Third School of Clinical Medicine (School of Rehabilitation Medicine), Zhejiang Chinese Medical University, Key Laboratory of Acupuncture and Neurology of Zhejiang Province, Hangzhou, China; ^2^ Department of Acupuncture, The Third Affiliated Hospital of Zhejiang Chinese Medical University, Hangzhou, China

**Keywords:** SCI, electroacupuncture, NLRP3, CMPK2, inflammasome

## Abstract

**Objectives:**

This study aimed to evaluate the expression of cytosine monophosphate kinase 2 (CMPK2) and activation of the NLRP3 inflammasome in rats with spinal cord injury (SCI) and to characterize the effects of electroacupuncture on CMPK2-associated regulation of the NLRP3 inflammasome.

**Methods:**

An SCI model was established in Sprague–Dawley (SD) rats. The expression levels of NLRP3 and CMPK2 were measured at different time points following induction of SCI. The rats were randomly divided into a sham group (Sham), a model group (Model), an electroacupuncture group (EA), an adeno-associated virus (AAV) CMPK2 group, and an AAV NC group. Electroacupuncture was performed at *jiaji* points on both sides of T9 and T11 for 20 min each day for 3 consecutive days. In the AAV CMPK2 and AAV NC groups, the viruses were injected into the T9 spinal cord *via* a microneedle using a microscope and a stereotactic syringe. The Basso–Beattie–Bresnahan (BBB) score was used to evaluate the motor function of rats in each group. Histopathological changes in spinal cord tissue were detected using H&E staining, and the expression levels of NLRP3, CMPK2, ASC, caspase-1, IL-18, and IL-1β were quantified using Western blotting (WB), immunofluorescence (IF), and RT-PCR.

**Results:**

The expression levels of NLRP3 and CMPK2 in the spinal cords of the model group were significantly increased at day 1 compared with those in the sham group (*p* < 0.05). The expression levels of NLRP3 and CMPK2 decreased gradually over time and remained low at 14 days post-SCI. We successfully constructed AAV CMPK2 and showed that CMPK2 was significantly knocked down following 2 dilutions. Finally, treatment with EA or AAV CMPK2 resulted in significantly increased BBB scores compared to those in the model group and the AAV NC group (*p* < 0.05). The histomorphology of the spinal cord in the EA and AAV CMPK2 groups was significantly different than that in the model and AAV NC groups. WB, IF, and PCR analyses showed that the expression levels of CMPK2, NLRP3, ASC, caspase-1, IL-18, and IL-1β were significantly lower in the EA and AAV CMPK2 groups compared with those in the model and AAV NC groups (*p* < 0.05).

**Conclusion:**

Our study showed that CMPK2 regulated NLRP3 expression in rats with SCI. Activation of NLRP3 is a critical mechanism of inflammasome activation and the inflammatory response following SCI. Electroacupuncture downregulated the expression of CMPK2 and inhibited activation of NLRP3, which could improve motor function in rats with SCI.

## Introduction

Spinal cord injury (SCI) is a devastating neurological condition that results in loss of motor and sensory functions (1) and is a major source of morbidity and mortality throughout the world (2). The overall global incidence of SCI was 10.5 cases per 100,000 individuals, resulting in an estimated 768,473 new cases annually worldwide (3). The number of individuals living with SCI is expected to increase with population growth, and SCI is expected to remain a considerable portion of the global injury burden (4–6). No treatments have been developed that promote functional recovery after injury (7). Improved understanding of the pathophysiology of SCI and evaluation of novel treatment strategies may benefit patients with SCI.

SCI is characterized by a primary injury caused directly by an initial trauma. The primary injury compromises neurons and glia, resulting in the initiation of a secondary injury cascade that leads to additional cell death and spinal cord damage over subsequent weeks (1, 8). Inflammation is one of the major barriers to neuronal anatomical and functional repair (9). After SCI, external stimulation and changes in the local microenvironment of the injury rapidly activate the immune system, thus activating the inflammatory response and promoting the release of IL-1β, IL-18, and other inflammatory factors (10). Inflammasome complexes are involved in the activation of caspase-1, which catalyzes the cleavage of pro-interleukins into their active forms (including IL-18 and IL-1β) (11–13). Studies have shown that the NLRP3 inflammasome plays a key role in this process (14–16). The NLRP3 inflammasome consists of PYD, NACHT, and LRR domains, which recruit the adaptor protein ASC and downstream caspase-1 through the PYD domain to form the inflammasome (17, 18). Jiang (19) reported that after SCI, drugs used to inhibit NLRP3 inflammasome activation reduced neuroinflammation and improved nerve recovery, which highlighted the importance of the NLRP3 inflammasome in secondary injury after SCI. Many recent studies have focused on exploring the mechanisms of NLRP3 inflammasome activation (20–23). However, the mechanisms by which SCI induces rapid NLRP3 activation and inflammation have not been characterized.

**Figure 1:**
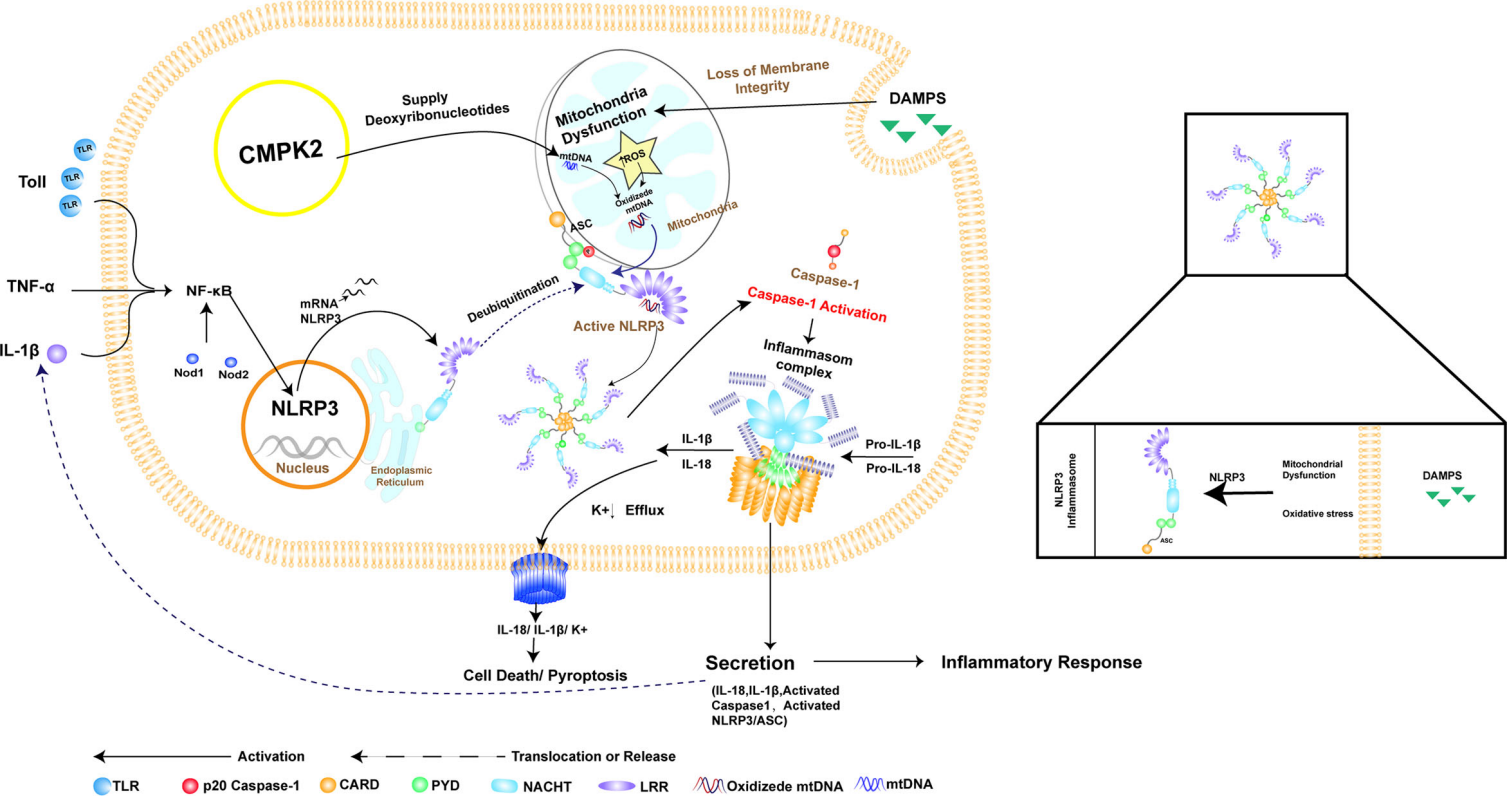
The relationship between NLRP3 inflammasome activation and CMPK2. NLRP3 consists of PYD, NACHT, and LRR domains, which recruit the joint protein ASC and downstream pro-caspase-1 to form the inflammasome. Assembly and activation of the NLRP3 inflammasome require mitochondrial damage, which results in the release of fragmented mtDNA and increased production of reactive oxygen species (ROS), resulting in oxidation of mtDNA (OX-mtDNA). Activation of the NLRP3 inflammasome involves pathogen-related or damage-associated molecular patterns (damPs), which are directly involved in toll-like receptor (TLR) activation, leading to rapid activation of NF-κB and induction of mitochondrial DNA (mtDNA) synthesis. Oxidized mtDNA is associated with the NLRP3 inflammasome complex. CMPK2 is a rate-limiting enzyme that provides deoxyribonucleotides for mtDNA synthesis, thus playing a key role in NLRP3 activation and the downstream inflammatory response. This graphic was generated using AI.

CMPK2 is an enzyme that catalyzes the transformation of nucleoside monophosphates into nucleoside diphosphates (24). Studies have shown that UDP, UTP, ADP, and ATP can activate macrophages through surface purinergic receptors to induce the expression of cytokines such as IL-1β (25). Furthermore, studies have shown that CMPK2 played a key role in NLRP3 activation, IL-1β production, and subsequent chronic inflammatory disease (26–30). However, the role of CMPK2 in NLRP3 inflammasome activation after SCI remains unclear (**Figure 1**).

In this study, we evaluated the expression of CMPK2 and NLRP3 in SCI rats. We successfully constructed adeno-associated virus (AAV) CMPK2 and showed that CMPK2 knockdown was most significant after 2× virus dilution. Finally, treatment with AAV CMPK2 resulted in significantly increased Basso–Beattie–Bresnahan (BBB) scores in SCI rats. The expression levels of CMPK2, NLRP3, ASC, caspase-1, IL-18, and IL-1β, as determined using Western blotting (WB), immunofluorescence (IF), and PCR, were decreased, and a similar effect was observed following electroacupuncture treatment, a widely used alternative therapy to treat SCI in China (31–34).

## Materials and Methods

### Reagent and Chemicals

A modified Model II-NYU/MASCIS impactor device for SCI modeling (W.M. Keck, USA) was purchased from the Key Laboratory of Acupuncture and Neurology of Zhejiang Province. The needle used for acupuncture was 0.25 mm × 25 mm and was purchased from Suzhou Medical Co., Ltd. (Jiangsu, China). The acupuncture point nerve stimulator was a HANS-200A from Huawei Co., Ltd. (Beijing, China). A Zeiss electric forward fluorescence microscope equipped with an Axio Imager M2 was purchased from Zeiss (Oberkochen, Germany). A Thermo NX50 cryostat was purchased from Thermo Fisher Scientific (Winsford, UK). The stereotaxic apparatus was purchased from RWD Life Science Co., Ltd. (Shenzhen, China). Mini-protean vertical electrophoresis and membrane transfer systems were purchased from Bio-Rad (Hercules, CA, USA). An Image Quant LAS4000 gel imaging system was purchased from GE Corporation (Frankfurt, Germany). A SpectraMax M4 microplate reader was purchased from MeiGu Molecular Co. Ltd. (Shanghai, China). Pentobarbital sodium and *N*,*N*,*N*′,*N*′-tetramethylethylenediamine (TEMED) were purchased from Sigma-Aldrich (St. Louis, MO, USA). Pierce™ BCA Protein Assay Kit was purchased from Thermo Fisher Scientific Inc. (Fair Lawn, NJ, USA). Difco™ skim milk was purchased from Becton, Dickinson and Company (Franklin Lakes, NJ, USA). Polyvinylidene fluoride (PVDF) membranes were purchased from Merck Millipore Ltd. (Billerica, MA, USA). Antibodies against NLRP3, CMPK2, ASC, and caspase-1 were purchased from Thermo Fisher. Antibodies against IL-18, IL-1β, and β-actin and Alexa Fluor 488-AffiniPure goat anti-rabbit were purchased from Abcam PLC (Cambridge, UK).

### Animals

Healthy adult male Sprague–Dawley (SD) rats (8 weeks old, 200–220 g of body weight) were purchased from the Shanghai Xipu Bikai Experimental Animal Company (animal license No. SCXK(Shanghai)2018-0006) and housed in the Laboratory Animal Center of Zhejiang Chinese Medical University, which is accredited by the Association for Assessment and Accreditation of Laboratory Animal Care (AAALAC, animal license No. SYXK(Zhejiang)2018-0012). Rats were maintained under controlled conditions with access to food and water *ad libitum*. All animal experiments were performed in compliance with all relevant ethical regulations for animal testing and research and in accordance with animal protocols approved by the animal ethics committee of Zhejiang Chinese Medical University (ZSLL, 2017-183). All experimental protocols strictly followed the guidelines of the National Institutes of Health (NIH) on the use of laboratory animals (NIH Publication No. 8023).

### Rat Spinal Cord Injury Model

**Figure 2:**
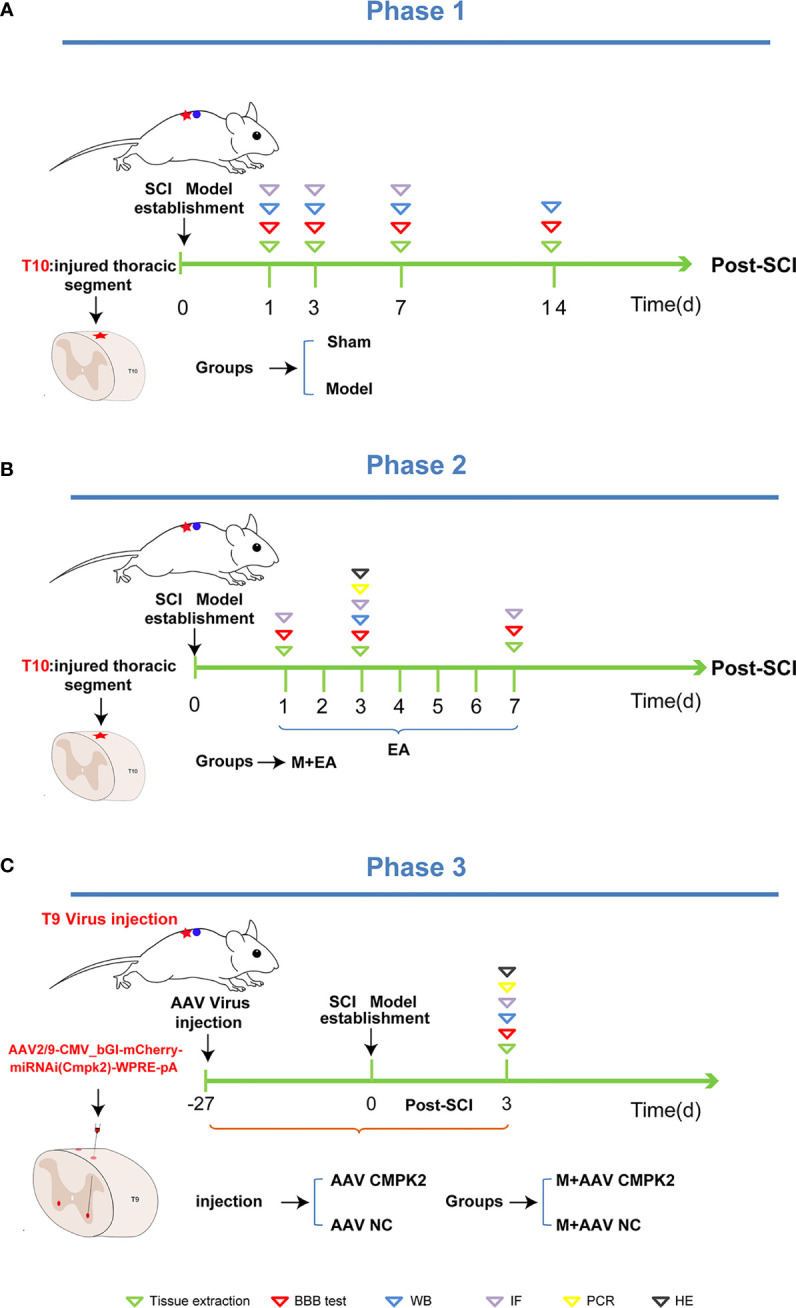
Phase 1, phase 2, and phase 3 of the experimental scheme. **(A)** Phase 1 is the establishment schedule of the spinal cord injury model. After T10 laminectomy, the rats were contused using a MASCIS weightless device with 5 × 10 g/cm gravitational potential energy. BBB scores were measured on days 1, 3, 7, and 14 post-SCI. The animals were then sacrificed, and tissues were extracted for Western blotting and immunofluorescence analysis. **(B)** Phase 2 is treatment with EA. After the establishment of the SCI model, EA intervention was administered on days 1, 3, and 7 post-SCI. We then measured the BBB score, extracted tissues, and performed Western blotting and immunofluorescence analyses. **(C)** Phase 3 is the establishment of the M+AAV CMPK2 and M+AAV NC groups. The SCI model was established in rats 27 days after injection of adeno-associated virus (AAV) using a stereolocator. The rats were evaluated to determine BBB score, and Western blotting, IF, PCR, H&E staining, and other analyses were performed. BBB, Basso–Beattie–Bresnahan; SCI, spinal cord injury; EA, electroacupuncture; IF, immunofluorescence.

To produce a contusive SCI model at T10, rats were placed on their ventral surface in a U-shaped stabilizer and then subjected to a T10 contusion using the MASCIS weight-drop device with a 5 × 10 g/cm gravitational potential energy after a T10 laminectomy. The severity and consistency of injury were verified by observing spinal cord congestion or tail lash of rats after impact, and BBB scores <3 were used as the standard for confirmation of successful model preparation. Rats in the sham group only underwent laminectomy. All animals were intraperitoneally injected with penicillin (100 U/day) for 3 consecutive days after modeling. After the procedure, the rats were returned to clean home cages that were partially placed on a heating pad until they fully recovered from anesthesia. Manual bladder expression was performed twice daily to promote bladder emptying (**Figure 2A**).

### Electroacupuncture Treatment

Electroacupuncture was performed on the T9–T11 *jiaji* (EX-B2) points on both sides of the spinous process on the backs of the rats. A disposable sterile stainless steel acupuncture needle with a diameter of 0.25 mm was inserted to a depth of 4–5 mm until the needle tip touched the lamina and was connected to a HANS-200A. The parameters were set as follows: the AC current (2/100 Hz) was applied on the first day after the operation, and the current intensity was maintained at 1 mA, causing slight muscle vibrations around the treatment area, for 20 min per day. Electroacupuncture treatment was divided into 3 time points and 3 subgroups, with treatment periods of 1, 3, or 7 consecutive days. Rats in the sham group, model group, and M+AAV CMPK2 group were tied in the prone position for 20 min but not subjected to EA (**Figure 2B**).

### Behavioral Testing

The BBB test is judged on a scale of 0–21 (0 = complete hind limb paralysis; 21 = normal locomotion) based on hind limb movements made in an open field including hind limb joint movement, weight support, plantar stepping, coordination, paw position, and trunk and tail control. The purpose of the BBB is to evaluate overall basic locomotor performance. Each rat was placed in an open field and evaluated for more than 3 min by two experimenters who were blinded to experimental groups, with one experimenter keeping count of the total score. All rats were assessed before modeling to ensure there were no baseline defects, and scores were averaged into a final score per session.

### CMPK2 Target Screening

To obtain effective interference targets against CMPK2, six CMPK2 targets of interfering plasmids WS0621–0626, overexpressing plasmid WS0588, and interfering control plasmid WX625 were cloned into the plasmid PAAV-CMV. Then, AAV 293T cells were cultured in Dulbecco’s modified Eagle’s medium and transfected to collect AAV-containing fractions. Then, the target sequence was screened using fluorescence attenuation and WB.

### Stereotaxic Injection of Viral Adeno-Associated Virus

To inhibit the expression of CMPK2, the rats were anesthetized with pentobarbital sodium (40 mg/kg, i.p.), and the hair on the back of T9 was removed using an electric shaver. The rats were fixed to a stereo-locater (RWD, 68025, Shenzhen, China), and a 10-μl Hamilton microsyringe was used to connect the rats to an ultramicro pump (WPI, UMC4, Sarasota, FL, USA) and its controller (WPI, UMC4, Sarasota, FL, USA). The rats were then injected with 0.5 μl of AAV2/9-CMV_bGI-mCherry-miRNAi (Cmpk2)-WPRE-pA (Shanghai Taitool Bioscience Co., Ltd., Shanghai, China) and negative control (AAV2/9-U6-shRNA(luciferase)-CAG-tdtomato virus) to the bilateral T9 spinal cord. After the T9 lamina was removed using forceps, the spinal cord was exposed with the median line as a reference, with a lateral opening of 1 mm and an inclination angle of 10°. The virus was injected under a microscope at a depth of 1.5 mm (**Figure 7A**). The virus was allowed to remain in the spinal cord for 5 min after the injection to avoid virus leakage. Blood vessels and nerves were avoided during injection, and the virus was diluted at a final titer of 2× before injection (7.8E + 12 v.g./ml) and infused at 100 nl/min, for a total of 550 nl/injection (**Figure 2C**).

### Immunofluorescence Staining

Rats were deeply anesthetized with sodium pentobarbital (50 mg/kg) and were perfused transcardially with 200 ml of 0.9% saline (4°C) followed by 200 ml of 4% formaldehyde. The ipsilateral T9–T11 spinal cord was harvested and post-fixed in the same fixative for 4 h (4°C) before being transferred to 15% and 30% sucrose for 72 h for dehydration. Several days later, the spinal cord was serially cut into 25-μm-thick transverse sections using a frozen microtome (Thermo NX50, USA) and mounted on gelatin-coated glass slides as 6 sets containing every fifth serial section. All slides were blocked with 5% normal goat serum in TBST (with 0.1% Tween-20) for 1 h at 37°C and then incubated overnight with the corresponding primary antibodies. The primary antibodies used were rabbit anti-NLRP3 (1:200, #PA5-79740, Thermo Fisher) and rabbit anti-CMPK2 (1:200, #PA5-34461, Thermo Fisher). The following day, the sections were rinsed with TBST (6 times, 10 min each) and incubated for 1 h with a mixture of corresponding secondary antibodies [Alexa Fluor 488-AffiniPure goat anti-rabbit IgG (H + L) (1: 600)]. Fluorescence images were captured using a Zeiss Structured Illumination Optical Section Microscope (Axio Imager M2). For quantitative fluorescence intensity analysis, uniform microscope settings were maintained throughout all image capture sessions. All stained sections were examined and analyzed in a blinded manner. Five images were randomly selected from each rat tissue, and positive cells were counted and averaged in the same area. Differences among groups were determined using one-way anOVA and two-way anOVA.

### Western Blotting

Rats were sacrificed on the third day after induction of SCI. The rats were deeply anesthetized using pentobarbital (50 mg/kg, i.p.) and transcardially perfused with 200 ml of normal saline (4°C). The spinal cord was immediately removed and stored at −80°C. Tissues were homogenized in radioimmunoprecipitation assay (RIPA) buffer [50 mm of Tris (pH 7.4),150 mM of NaCl, 1% Triton X-100, 1% sodium deoxycholate, sodium orthovanadate, 0.1% sodium dodecyl sulfate (SDS), EDTA, sodium fluoride, leupeptin, and 1 nM of phenylmethylsulfonyl fluoride (PMSF)] and then centrifuged at 15,000 rpm for 15 min at 4°C, after which the supernatant was collected. The protein concentration was determined using the bicinchoninic acid (BCA) method according to the manufacturer’s instructions (Thermo Fisher, USA), and 20 μg of protein was loaded in each lane. Protein samples were separated on 8%–12% SDS–polyacrylamide gel electrophoresis (SDS-PAGE) gels and electrophoretically transferred to PVDF membranes (Merck KGaA, Darmstadt, Germany). The membranes were blocked with 5% non-fat milk at room temperature for 1 h and then incubated at 4°C overnight with the following primary antibodies diluted in blocking buffer: NLRP3, CMPK2, caspase-1, ASC, IL-1β, IL-18, and β-actin. The next day, the membrane was incubated with the secondary antibody (1:5,000, #7074, CST, Danvers, MA, USA) for 2 h at room temperature. Immunoreactivity was detected using enhanced chemiluminescence and visualized using an Image Quant LAS 4000. The density of each band was measured using Image Quant TL 7.0 analysis software. The relative expression of the target protein is (target protein absorbance value)/(actin absorbance value), and the results are expressed as the mean ± standard deviation.

### H&E Staining

The rats in each group were anesthetized by intraperitoneal injection of 3% sodium pentobarbital (50 mg/kg) at different time points. After intubation in the left ventricle, the right auricle was cut open and rinsed with a rapid infusion of normal saline. Once the outflow liquid was clear, 40 g/L of paraformaldehyde was slowly infused through the heart. The spinal cord was quickly cut 4 cm from the injury center and fixed in 4% paraformaldehyde for 12 h. After post-fixation, gradient alcohol dehydration, and paraffin embedding, continuous transverse sections were prepared, H&E staining and resin sealing were performed, and images were collected using a digital pathological section scanning system (C13210-01, Hamamatsu Photonics, Hamamatsu, Japan).

### Reverse Transcriptase PCR

Total RNA was extracted from the spinal cord tissue using TRIzol reagent (Invitrogen, Carlsbad, CA, USA) according to the manufacturer’s protocol. Primer sequences are listed in **Table 1**. Quantitative PCR was performed using the Fast Start Universal SYBR Green Master kit (TaKaRa Bio Inc., Dalian, China) according to the manufacturer’s protocol on a CFX96 Real-Time System (Bio-Rad, Hercules, CA, USA). Each reaction was performed in triplicate and normalized to GAPDH gene expression. The CT value of each well was determined using the CFX96 Real-Time System software, and the average of the triplicates was calculated. Relative quantification was determined using the 2^−ΔΔCT^ method.

**Table 1:** Sequences of the primers used for qPCR.

Ranking	Sequence name	Primer sequence (5′ to 3′)	Amplicon size (bp)
1	NLRP3	F:5′-GAGCTGGACCTCAGTGACAATGC-3′	146
		R:5′-ACCAATGCGAGATCCTGACAACAC-3′	
2	CMPK2	F:5′-TGCCCGATTGCTCCCTGACTC-3′	131
		R:5′-GCCTTCGCCTGGAACCAATGG-3′	
3	Caspase-1	F:5′-GTGGTTCCCTCAAGTTTTGC-3′	154
		R:5′-CCGACTCTCCGAGAAAGATG-3′	
4	ASC	F:5′-GGAGGGGTATGGCTTGGAG-3′	179
		R:5′-TGAGTGCTTGCCTGTGTTGGT-3′	
5	IL-18	F:5′-ATATCGACCGAACAGCCAAC-3′	105
		R:5′-TTCCATCCTTCACAGATAGGG-3′	
6	IL-1β	F:5′-CAACTGTTCCTGAACTCAACTG-3′	281
		R:5′-GAAGGAAAAGAAGGTGCTCATG-3′	

### Statistical Analysis

GraphPad Prism 8.0 was used to conduct statistical analyses. The data in the figures are expressed as the mean ± SEM. One-way or two-way ANOVA followed by Tukey’s *post hoc* test was used for comparisons among groups ≥5. Comparisons were considered significantly different when *p* < 0.05.

## Results

### Motor Dysfunction after Spinal Cord Injury

**Figure 3:**
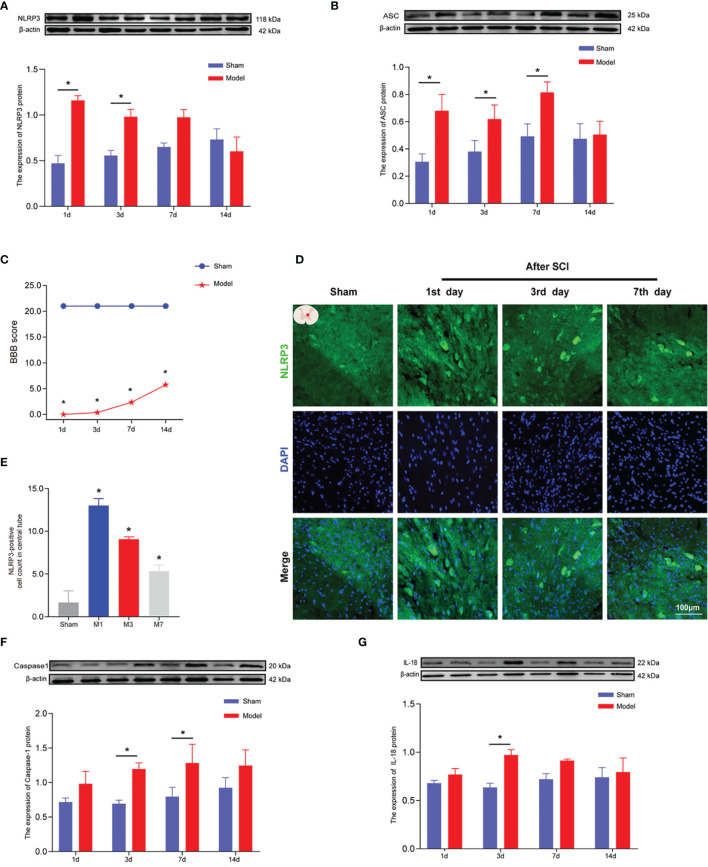
The expression levels of NLRP3, ASC, caspase-1, and IL-18 in rat spinal cords 1, 3, 7, and 14 days after SCI. **(A)** Representative Western blotting and semiquantitative analysis of NLRP3 protein in the spinal cords of rats in the sham and model groups at days 1, 3, 7, and 14 post-SCI. The image shows representative Western blotting for NLRP3 and β-actin. **(B)** Western blotting was used to analyze the protein expression of ASC in the sham and model groups after SCI. Representative protein bands are shown for ASC at 1, 3, 7, and 14 days after SCI (N = 5 rats/group). The expression of ASC is shown for the model and sham groups. *p < 0.05; two-way ANOVA. **(C)** The BBB function scores in the sham and model groups. N = 8 rats/group. *p < 0.05. **(D)** The expression of NLRP3 in the spinal cords of rats was detected using IF. The expression of NLRP3 in the spinal cord was detected in the sham group and 1, 3, and 7 days after SCI. Cells positive for NLRP3 protein are shown in green. **(E)** Quantitative analysis of NLRP3-positive cells in the model group compared with the sham group. *p < 0.05; one-way ANOVA was used for comparison. N = 5 rats/group. **(F, G)** Protein expression and representative protein bands for caspase-1 and IL-18 at each time point after SCI as determined using Western blotting. N = 5 rats/group. *p < 0.05; two-way ANOVA was used for comparison. Data are presented as the mean ± SEM. SCI, spinal cord injury; BBB, Basso–Beattie–Bresnahan; IF, immunofluorescence.

**Figure 4:**
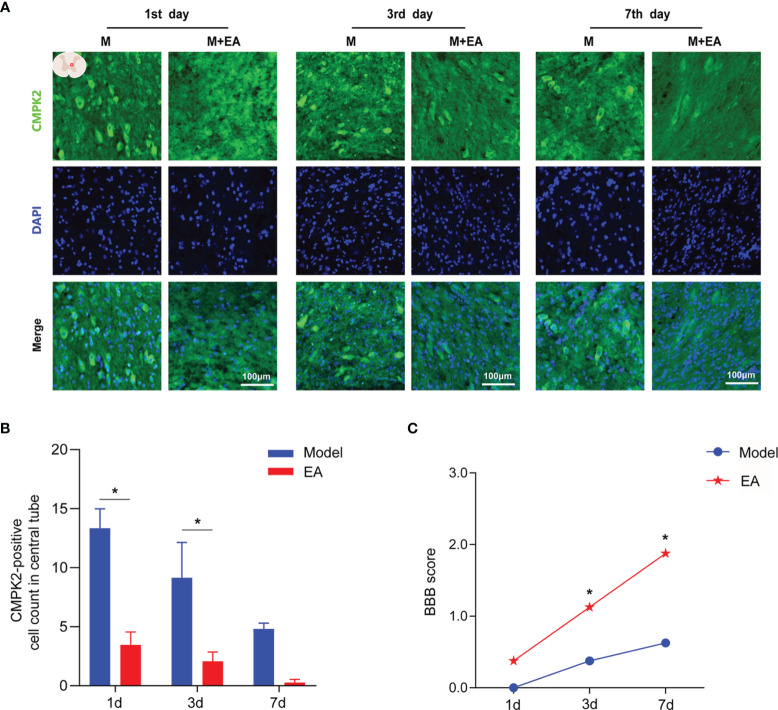
Immunofluorescence analysis showed positive expression of CMPK2 in spinal cord tissues of the model and EA groups. **(A)** Immunofluorescence labeling represented the number of CMPK2-positive cells (green fluorescence) at 1, 3, and 7 days after SCI, and the CMPK2-positive protein staining area is shown in green. **(B)** Quantitative analysis of CMPK2-positive cells. *p < 0.05 compared with the model group; one-way anOVA was used for comparison. N = 5 rats/group. **(C)** BBB scores for the model and EA groups. N = 8 rats/group. *p < 0.05 compared with the sham group. Data are presented as the mean ± SEM. EA, electroacupuncture; BBB, Basso–Beattie–Bresnahan.

We generated a T10 contusion model of sci, as shown in **Figure 2A**, and BBB scores were used to evaluate a motor function on days 1, 3, 7, and 14 after SCI. The BBB scores began to rise on the second day, and motor function gradually improved, with the highest BBB scores achieved on day 14 after SCI, as evidenced by the significant recovery of hind limb function (**Figure 3C**). After T10 contusion SCI, we continuously administered EA for 3 days. The BBB scores in the EA group began to increase on the third day and showed a significantly higher rate of increase than that in the model group (**Figure 4C**). The results showed that hind limb motor function was significantly impaired following SCI. Although motor function recovered slightly following injury, the degree of improvement was greater following EA intervention than that in the model group.

### Activation of the NLRP3 Inflammasome After Spinal Cord Injury

Motor function scores were measured 1, 3, 7, and 14 days after SCI (**Figure 3C**). The BBB score results showed that the model group had significantly impaired motor function compared with the sham group (*p < 0.05). Hind limb motor function began to recover on the 3rd day after SCI, and BBB score was the highest on the 14th day of observation. WB (**Figures 3A, B, F, G** **)** was used to measure the expression levels of NLRP3, ASC, caspase-1, and IL-18 at 1, 3, 7, and 14 days after SCI to determine the timing of inflammatory mediator expression. The protein expression levels of NLRP3 in the model group were greater than those in the sham group and were significantly increased at 1 and 3 days after SCI (*p* < 0.05; **Figure 3A**). After SCI, the number of NLRP3-positive cells (marked in green) around the central canal of the spinal gray matter increased significantly (**Figures 3D, E** **)**. IF results were consistent with those obtained using WB. At 1, 3, and 7 days after SCI, the expression of ASC protein in the spinal cord tissue of rats in the model group was significantly increased (**Figure 3B**) compared with that in the sham group (*p* < 0.05). On days 3 and 7 after SCI, the expression of caspase-1 in the spinal cord tissue of rats in the model group was significantly increased (**Figure 3F**) compared with that in the sham group (*p* < 0.05). Three days after SCI, the expression of IL-18 in the spinal cords of rats in the model group was significantly increased (**Figure 3G**) compared with that in the sham group (*p* < 0.05).

### Expression of CMPK2 in Spinal Cord After Spinal Cord Injury

**Figure 5:**
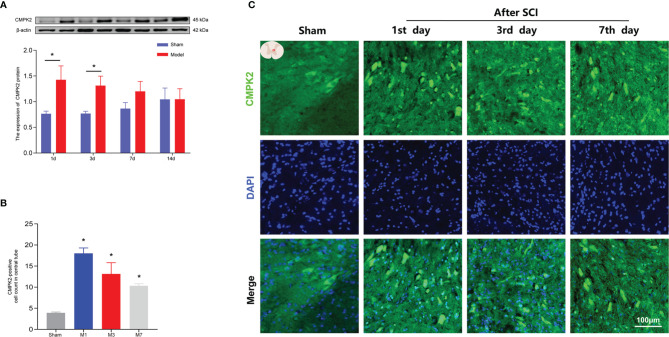
The expression of CMPK2 in rat spinal cords was detected using WB and IF. **(A)** Western blotting was used to analyze the protein expression of CMPK2 in the sham and model groups after SCI. Representative protein bands are shown for CMPK at 1, 3, 7, and 14 days after SCI (N = 5 rats/group). *p < 0.05; two-way ANOVA was used for comparison. **(B)** Quantitative analysis of CMPK2-positive cells. *p < 0.05 compared with the sham group; one-way anOVA was used for comparison. N = 5 rats/group. **(C)** CMPK2 was expressed around the central canal of the gray matter of the spinal cord at 1, 3, and 7 days after spinal cord injury. CMPK2-positive cells are represented by green fluorescence. DAPI (blue) co-staining was used to identify positive cells. Data are presented as the mean ± SEM. WB, Western blotting; IF, immunofluorescence; SCI, spinal cord injury.

Recent studies have shown that CMPK2 plays a key role in NLRP3 activation and chronic inflammatory disease (9, 10). To confirm that CMPK2 plays an important role in NLRP3 activation, WB and IF were performed to evaluate CMPK2 expression. Compared with that in the sham group, the expression of CMPK2 protein in the model group was significantly increased on days 1 and 3 post-SCI (**Figure 5A**). The IF results were consistent with the results obtained using WB. After SCI, the expression of CMPK2 (marked in green) around the central canal of the spinal gray matter increased significantly (**Figures 5B, C** **)**, which indicated that CMPK2 was significantly upregulated around the central tubules of the spinal gray matter at days 1, 3, and 7 after SCI.

### Effect of Electroacupuncture on CMPK2 Expression in Spinal Cord Injury Rat Spinal Cords

To observe the effect of EA on the expression of CMPK2 around the central canal of the spinal cord at 1, 3, and 7 days after SCI, we performed IF and determined BBB scores for rats in the EA and model groups. The BBB scores were significantly higher in the EA group than those in the model group at 3 and 7 days post-SCI (*p* < 0.05) (**Figure 4C**). IF results showed that EA intervention significantly reduced the number of CMPK2-positive cells (green fluorescence) compared with that in the model group at 1 and 3 days post-SCI (**Figures 4A, B** **;** *p* < 0.05).

### CMPK2 Target Screening

**Figure 6:**
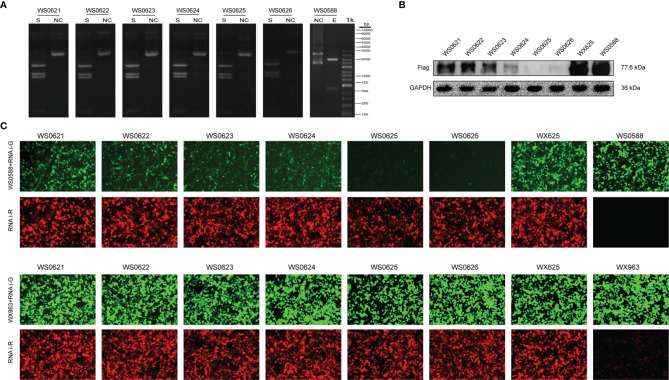
*In vitro* CMPK2 target screening. **(A)** Plasmid digestion validation for WS0588 and WS0621-0626 using the following restriction sites: *Sma*l (S) and *Kpn*I–*Bam*HI (E). Molecular weight was determined using a 1-kb DNA Ladder (from bottom to top: 100 bp, 250 bp, 500 bp, 750 bp, 1 kb, 2 kb, 3 kb, 4 kb, 5 kb, 6 kb, 8 kb, and 10 kb). The pictures in panel A represent each plasmid treated with restriction enzyme (S) and unrestricted controls (NC). The restriction site was *Sma*l for WS0621, WS0622, WS0623, WS0624, WS0625, and WS0626, and the restriction site of WS0588 was *Kpn*I–*Bam*HI. The size of each band corresponded with the expected value. **(B)** Western blotting of the interference group WS0621–626, the interference control sample WX625, and the overexpression sample WS0588 Western blotting bands. The flag signal was present at 77.6 kDa. WS0625 and WS0626 proteins were least expressed, which indicated the best interference effect. **(C)** The interference group WS0621–626, the interference control sample WX625, and the overexpression sample WS0588 were co-transfected with 293 T cells *in vitro*. Red fluorescence represents the expression of interfering plasmids. Green fluorescence represents overexpression of the target gene plasmid. In the experimental group, the green fluorescence was significantly reduced for the WS0625 and WS0626, which indicated that these targets had strong interference ability. The negative control WX625 had no knockout ability. The green fluorescence in the interference control sample WX625 and overexpression sample WS0588 were significantly enhanced.

**Figure 7:**
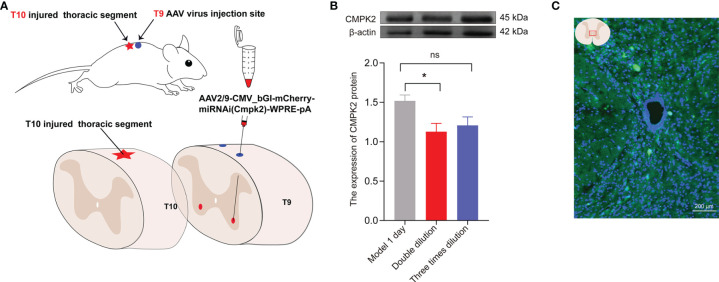
The expression of CMPK2 around the central canal of the spinal cord was decreased after CMPK2 knockdown. **(A)** Schematic diagram of virus injection. **(B)** Quantitation of CMPK2 in the T10 spinal cord 4 weeks after virus injection using Western blotting. The results show CMPK2 protein expression after 2× and 3× viral dilutions. N = 3 rats/group. *p < 0.05 compared with the model group; one-way anOVA was used for comparison. **(C)** Fluorescent representation of CMPK2 expression in the T10 spinal cord 4 weeks after virus injection. All data are expressed as the mean ± SEM.

To further verify the relationship between CMPK2 and NLRP3 after SCI, we designed 6 groups of miRNAi sequence plasmids with green fluorescent protein (GFP) fluorescence labels, which were integrated into AAV and then transfected into HEK293 cells to observe the degree of translation. In this study, 293T cells were co-transfected with interference group WS0621–626, interference control sample WX625, and overexpression sample WS0588 *in vitro* using the fluorescence attenuation test. The negative control, WX625, which was found to have interference targets, did not result in a significant knockdown. In contrast, the WS0625 and WS0626 target sequences resulted in substantial interference (**Figure 6C**). WB showed that the protein expression levels in the WS0625 and WS0626 groups were low, which indicated that the two targets had good interference effects (**Figures 6A, B**). Therefore, WS0625 and WS0626 were the best sequences for use as inhibitors (**Table 2**). As a result, WS0626 was selected to package AAV2/9-CMV_bGI-mCherry-miRNAi (Cmpk2)-WPRE-pA for subsequent experiments. To verify the knockdown efficiency of CMPK2 in rats, AAV2/9-CMV_bGI-mCherry-miRNAi (Cmpk2)-WPRE-pA was injected into the T9 spinal cord for 27 days before modeling using stereometric microinjection. However, injection with the original virus titer (1.56E + 13 v.g./ml) resulted in increased mortality. Therefore, we diluted the original virus and found that virus knockdown efficiency was best after 2× dilution (**Figures 7B, C**), as evidenced by significantly reduced CMPK2 expression in the knockdown group compared with that in the model group (*p* < 0.05). An AAV2/9-U6-shRNA(luciferase)-CAG-tdtomato blank virus was used as a control. Samples were extracted for WB and IF analyses on day 3 after SCI. The results showed that AAV2/9-CMV_bGI-mCherry-miRNAi (Cmpk2)-WPRE-pA diluted 2× significantly reduced the expression of CMPK2 after SCI.

**Table 2:** CMPK2 target sequence.

Ranking	Target sequence	Plasmid
1	GCTGAGCAAACTGCTGGGATA	WS0621
2	TCTTGGAGGAGTGCACATCTT	WS0622
3	GTCAGAGTCTCTCCAAGCTGT	WS0623
4	GTGGAGGAAGCTCTTTGATGA	WS0624
5	GCTAAACAGTCAGCCAAGTTT	WS0625
6	GGTCAACAGCGTGTTTCGTCA	WS0626

### Effects of Electroacupuncture and Adeno-Associated Virus on CMPK2 Expression in Spinal Cord Injury Rats

**Figure 8:**
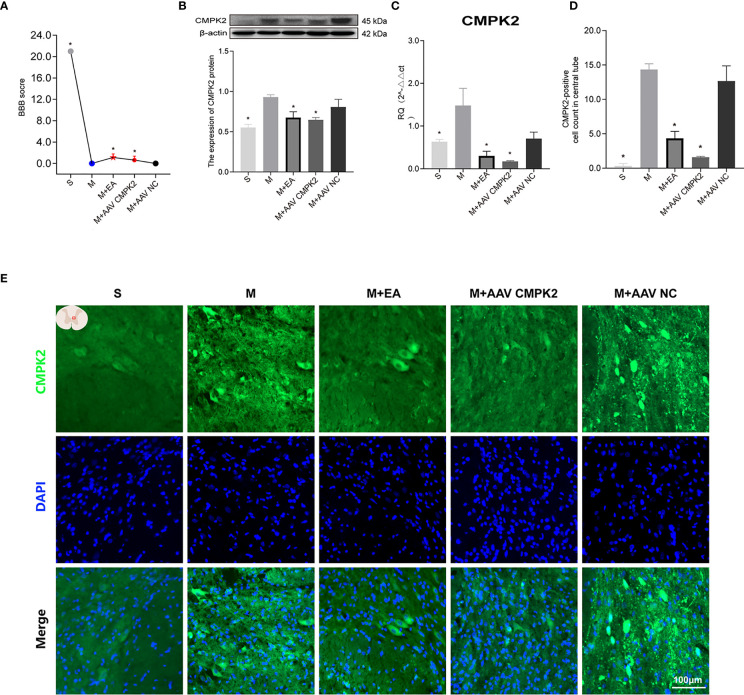
Effect of EA on CMPK2 expression in spinal cords of rats following CMPK2 knockdown. **(A)** BBB scores of rats in the Sham, Model, M+EA, M+AAV CMPK2, and M+AAV NC groups. N = 8. *p < 0.05 compared with the model group. **(B)** Western blotting analysis of CMPK2 expression in spinal cord tissues of rats in the Sham, Model, M+EA, M+AAV CMPK2, and M+AAV NC groups. A representative Western blotting and semiquantitative analysis of CMPK2 are shown. N = 5 rats/group. *p < 0.05 compared with the model group; one-way anOVA was used for comparison. **(C)** Evaluation of CMPK2 gene expression using qPCR in the Sham, Model, M+EA, M+AAV CMPK2, and M+AAV NC groups 3 days after SCI. N = 5 rats/group. *p < 0.05 compared with the model group; one-way anOVA was used for comparison. **(D)** Quantitative analysis of CMPK2-positive cells. *p < 0.05 compared with the model group; one-way anOVA was used for comparison. N = 5 rats/group. **(E)** Immunofluorescence was used to detect the expression of CMPK2 around the central canal of the spinal gray matter of rats in the Sham, Model, M+EA, M+AAV CMPK2, and M+AAV NC groups. Number of CMPK2-positive cells (green fluorescence) 3 days after SCI. DAPI (blue) co-staining was used to identify positive cells. All data are expressed as the mean ± SEM. EA, electroacupuncture; BBB, Basso–Beattie–Bresnahan; SCI, spinal cord injury.

The expression of CMPK2 and activation of the NLRP3 inflammasome were evaluated after intervention with EA or AAV. To demonstrate the relationship between NLRP3 and CMPK2, we used AAV2/9-CMV_bGI-mCherry-miRNAi (Cmpk2)-WPRE-pA, an effective AAV (designed and customized by Shanghai Tierto Biotechnology Co., Ltd., Shanghai, China) for knockdown of CMPK2 expression. In this experiment, BBB score was used to evaluate hind limb motor function in each group after CMPK2 knockdown. The results showed that the BBB score decreased from 21 prior to SCI to 0 at day 3 post-SCI. Intervention with EA or AAV virus resulted in a BBB score of 3 at day 3 post-SCI, which was significantly higher than that observed in the model group (*p < 0.05) (**Figure 8A**). WB and IF showed that the expression of CMPK2 protein increased after SCI (**Figures 8B, E**) in the model and M+AAV NC groups. In contrast, the expression of CMPK2 protein was significantly lower in the EA and M+AAV CMPK2 groups. Three days after SCI, WB and IF results showed significant differences between the model group and the sham, M+EA, and M+AAV CMPK2 groups (*p* < 0.05) (**Figures 8B, D**). The results of the analysis of CMPK2 using PCR (**Figure 8C**) were consistent with those obtained using WB and IF.

### Effects of Electroacupuncture and Adeno-Associated Virus Intervention on Activation of NLRP3 Inflammasome in Spinal Cord Injury Rats

**Figure 9:**
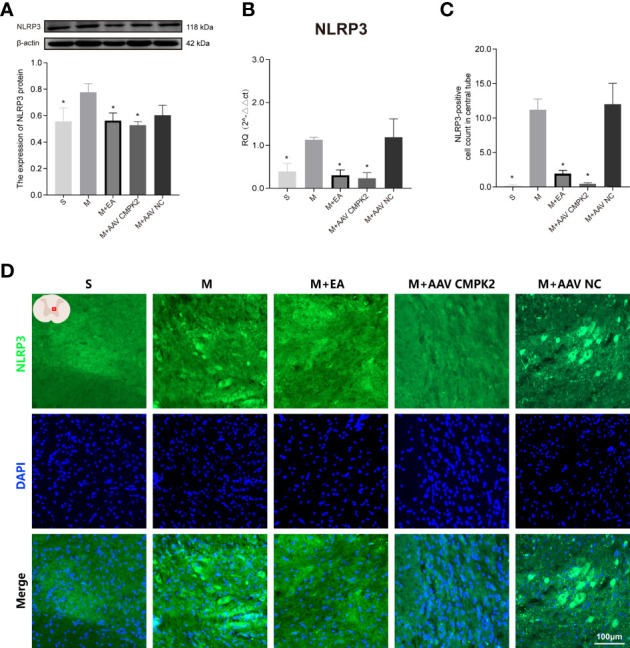
Effects of CMPK2 knockdown on activation of the NLRP3 inflammasome in rat spinal cord. **(A)** Western blotting analysis of NLRP3 expression in spinal cords of rats in the Sham group, Model, M+EA, M+AAV CMPK2, and M+AAV NC groups. A representative Western blotting and semiquantitative analysis of NLRP3 protein levels are shown. N = 5 rats/group. *p < 0.05 compared with the Model group; one-way anOVA was used for comparison. **(B)** Quantitative PCR analysis of NLRP3 gene expression in the spinal cords of rats in the Sham, Model, M+EA, M+AAV CMPK2, and M+AAV NC groups. N = 5 rats in each group. *p < 0.05 compared with the model group; one-way anOVA was used for comparison. **(C)** Quantitative analysis of NLRP3-positive cells. *p < 0.05 compared with the model group; one-way anOVA was used for comparison. N = 5 rats/group. **(D)** The expression of NLRP3 in the spinal cords of rats in the Sham, Model, M+EA, M+AAV CMPK2, and M+AAV NC groups were detected using IF. The number of CMPK2-positive cells (green fluorescence) 3 days after SCI was labeled by immunofluorescence, and the areas with positive NLRP3 staining are shown in green. All data are expressed as the mean ± SEM. IF, immunofluorescence; SCI, spinal cord injury.

**Figure 10:**
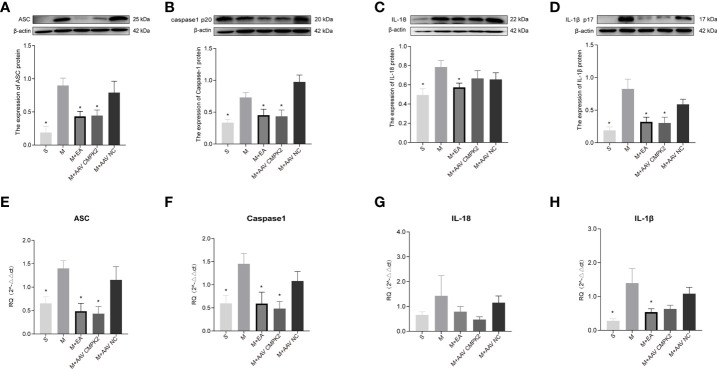
Expression of ASC, caspase-1, IL-18, and IL-1β in spinal cords of rats after CMPK2 knockdown. **(A–D)** Western blotting detection of ASC, caspase-1, IL-18, and IL-1β expression in the spinal cords of rats in the Sham, Model, M+EA, M+AAV CMPK2, and M+AAV NC groups showed increased expression of all inflammatory complexes in the Model group. Intervention with EA or AAV virus reversed this increase. Representative images show ASC, caspase-1, IL-18, IL-1β, and β-actin protein expression. Semiquantitative analysis of ASC, caspase-1, IL-18, and IL-1β is shown for each group at 3 days post-SCI. N = 5 rats/group. *P < 0.05 compared with the model group. One-way anOVA was used for comparison. **(E**–**H)** Gene expression of ASC, caspase-1, IL-18, and IL-1β in the spinal cords of rats in the Sham, Model, M+EA, M+AAV CMPK2, and M+AAV NC groups using qPCR. N = 5 rats in each group. All data are expressed as the mean ± SEM. AAV, adeno-associated virus; EA, electroacupuncture.

Following confirmation that EA and CMPK2 knockdown reduced CMPK2 expression in spinal cords of SCI rats, WB, IF, and PCR results showed that NLRP3 inflammasome protein expression significantly increased after SCI (**Figures 9A–D**). SCI resulted in increased expression of ASC, caspase-1, IL-18, and IL-1β, and treatment with EA reversed these increases (**Figures 10A–D**). Analysis of gene expression levels of ASC, caspase-1, IL-18, and IL-1β using PCR agreed with the results obtained using WB (**Figures 10E–H**). Three days after SCI, WB and IF results showed that NLRP3 protein expression in the spinal cords of rats in the sham, M+EA, and M+CMPK2 AAV groups was significantly decreased compared with that in the model group (*p* < 0.05; **Figures 9A, C** **)**.

### Changes in H&E Staining in the Spinal Cord

**Figure 11:**
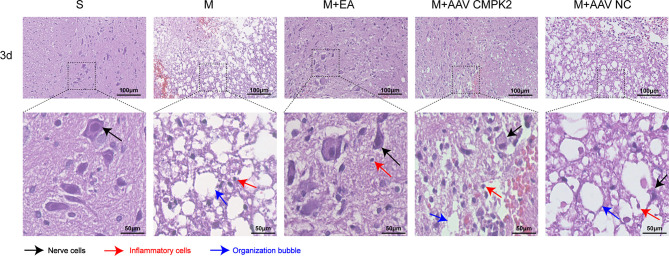
Pathological examination results of H&E staining of rat spinal cord tissues in each group. Representative images show the spinal cord around the central canal 3 days after SCI in the sham, model, M+EA, M+AAV CMPK2, and M+AAV NC groups. The black arrows indicate nerve cells. The red arrows indicate inflammatory cells. Blue arrows indicate tissue vacuoles. N = 3 rats/group. SCI, spinal cord injury.

To observe the pathological changes of the area surrounding the SCI in the sham, model, M+EA, M+AAV CMPK2, and M+AAV NC groups at 3 days post-SCI, H&E staining was performed on 3 rats in each of the 5 groups. The spinal cord tissue structure in the sham group (S) was complete, the gray and white matter boundaries were clear, nuclear morphology was normal, nucleoli were large and clear, and there was no evidence of inflammatory cell infiltration, hemorrhage, necrosis, or tissue edema. The model (M) and AAV virus (M+AAV NC) groups showed obvious patches of bleeding, loose tissue, formation of a large number of vacuoles, obvious changes in cell morphology, cell body shrinkage, nuclear pyknosis, and increased numbers of inflammatory cells in the spinal cord 3 days after SCI. In contrast, the spinal cord tissue structure in the EA and knockdown virus (M+AAV CMPK2) groups was relatively complete compared with that in the model and AAV NC groups, with small bleeding foci, reduced tissue vacuoles, increased numbers of nerve cells, and decreased numbers of inflammatory cells (**Figure 11**).

## Discussion

Excessive inflammation following SCI is a major obstacle to neurological recovery. The NLRP3 inflammasome plays a central role in many acute and chronic inflammatory and degenerative diseases (35–38), but the mechanisms that control its activation are poorly understood. Recent studies have confirmed that CMPK2 plays a key role in NLRP3 activation, IL-1β production, and subsequent chronic inflammatory disease (26, 27). We examined CMPK2 expression and NLRP3 inflammasome activation following SCI. Studies have shown that NLRP3 consists of PYD, NACHT, and LRR domains, which recruit the adaptor protein ASC and caspase-1 through the PYD domain, resulting in inflammasome activation and increased release of IL-18 and IL-1β. In our study, the expression levels of NLRP3, ASC, caspase-1, and IL-18 were significantly increased at the beginning of day 1 post-SCI, which indicated that the NLRP3 inflammasome was activated following SCI. These results were consistent with the current clinical approach to intervene as quickly as possible following SCI. These results also confirmed that NLRP3 inflammasome activation after SCI can contribute to secondary injury (39, 40). We also showed that the expression of CMPK2 increased after SCI, and the expression levels of inflammatory factors increased in parallel with the expression of CMPK2. These increases were accompanied by decreases in BBB scores. Intervention with electroacupuncture reversed SCI-induced increases in expression levels of NLRP3 and CMPK2, reduced accumulation of NLRP3 and CMPK2 around the central gray matter, promoted the recovery of motor function, and increased BBB score. These findings indicated that CMPK2 may be closely related to activation of the NLRP3 inflammasome.

To further investigate the correlation between NLRP3 and CMPK2 in spinal cord tissue of rats after SCI, we screened CMPK2 target sequences *in vitro* and packaged them into viruses for injection into the T9 spinal cord. The results showed that injection with AAV CMPK2, which effectively knocked down CMPK2, significantly improved motor function in rats following SCI compared with the model and AAV NC (control virus) groups. This improvement further supported the role of CMPK2 in the motor function deficits associated with SCI. Furthermore, CMPK2 knockdown inhibited activation of the NLRP3 inflammasome, which confirmed that CMPK2 was involved in NLRP3 inflammasome activation. Confirmation of the association between CMPK2 and the NLRP3 inflammasome was consistent with previous studies that showed that CMPK2 was closely associated with NLRP3 inflammasome activation in hepatic ischemia/reperfusion (I/R) injury and acute respiratory distress syndrome (ARDS), which may inhibit the synthesis of ATP and mtDNA (30, 41).

Electroacupuncture has been used as an alternative treatment for various diseases in recent years (42–44). A number of studies have shown that EA can improve motor function in the lower limbs of patients with SCI, resulting in improved quality of life (32–34). However, the mechanism by which EA confers therapeutic benefit is unclear. We previously showed that EA intervention regulated the inflammatory response, and EA at the *jiaji* point promoted the expression of the transcription factors Olig2 and Sox10 after SCI, which promoted proliferation and differentiation of oligodendrocyte precursor cells into oligodendrocytes, resulting in the recovery of motor function in rats (34, 45). In this study, the expression levels of NLRP3, CMPK2, ASC, caspase-1, and IL-18 were significantly decreased after EA treatment or CMPK2 knockdown, which indicated that these treatments inhibited activation of the NLRP3 inflammasome. Furthermore, EA and CMPK2 knockdown each resulted in improved BBB scores and histomorphological changes. These results further confirmed that EA can mitigate the inflammatory response following SCI by influencing CMPK2 expression and inhibiting NLRP3 inflammasome activation.

## Conclusions

Our study showed that CMPK2 can regulate NLRP3 expression in rats with SCI. Activation of NLRP3 is an important component of the inflammatory response following SCI. In addition, EA reduced the expression of CMPK2, which inhibited activation of the NLRP3 inflammasome, reduced inflammation, and preserved motor function.

## Data Availability Statement

The raw data supporting the conclusions of this article will be made available by the authors, without undue reservation.

## Ethics Statement

The animal study was reviewed and approved by ZSLL, 2017-183.

## Author Contributions

RM and LW designed this study. LW, YC, DZ, and MS completed this study. YC, DZ, and HX participated in the modeling of spinal cord injury. YC and XW performed electroacupuncture treatment. YC and RH analyzed the data. LW, YC, SH, and KH wrote the manuscript. XMS participated in the revision of the article. RM validated the manuscript. All authors contributed to the article and approved the submitted version.

## Funding

The project was supported by the project funding of the Zhejiang Provincial Natural Science Foundation of China (No. LQ21H270003 to LW), Zhejiang Chinese Medical University Research Fund (2018ZG18; KC201945; 2019ZG17; 2020YKJ08), the Chinese Medicine Research Program of Zhejiang Province (No. 2019ZZ013 to RM), and the National Natural Science Foundation of China (No. 82174487 to RM). The Third Clinical College of Zhejiang Chinese Medical University provided the experimental site and instruments for this experiment.

## Conflict of Interest

The authors declare that the research was conducted in the absence of any commercial or financial relationships that could be construed as a potential conflict of interest.

## Publisher’s Note

All claims expressed in this article are solely those of the authors and do not necessarily represent those of their affiliated organizations, or those of the publisher, the editors and the reviewers. Any product that may be evaluated in this article, or claim that may be made by its manufacturer, is not guaranteed or endorsed by the publisher.

## Abbreviations

SCI, spinal cord injury; EA, electroacupuncture; BBB, Basso–Beattie–Bresnahan; WB, Western blotting; IF, immunofluorescence; CMPK2, cytosine monophosphate kinase 2; TEMED, *N*,*N*,*N*′,*N*′-tetramethylethylenediamine; ROS, reactive oxygen species; TLR, toll-like receptors; mtDNA, mitochondrial DNA; AAV, adeno-associated virus; I/R, ischemia/reperfusion; ARDS, acute respiratory distress syndrome.
